# Symptom burden, healthcare utilization, and risky behaviors in survivors of the childhood cancer survivor study (CCSS): an observation cohort study

**DOI:** 10.1016/j.eclinm.2025.103657

**Published:** 2025-11-20

**Authors:** Rachel Webster, Deo Kumar Srivastava, Lu Xie, Himani Darji, Wei Liu, Meghan E. McGrady, Tara M. Brinkman, Nicole M. Alberts, Kirsten K. Ness, Bernard Fuemmeler, Alicia S. Kunin-Batson, I-Chan Huang, Gregory T. Armstrong, Rebecca M. Howell, Daniel M. Green, Yutaka Yasui, Kevin R. Krull

**Affiliations:** a Department of Psychology and Biobehavioral Sciences, St. Jude Children's Research Hospital, Memphis, TN, USA; b Comprehensive Cancer Center, St. Jude Children's Research Hospital, Memphis, TN, USA; c Department of Biostatistics, St. Jude Children's Research Hospital, Memphis, TN, USA; d Phastar Inc., Cambridge, MA, USA; e Division of Behavioral Medicine and Clinical Psychology, Cincinnati Children's Hospital Medical Center, Cincinnati, OH, USA; f Department of Pediatrics, University of Cincinnati College of Medicine, Cincinnati, OH, USA; g Department of Epidemiology and Cancer Control, St. Jude Children's Research Hospital, Memphis, TN, USA; h Department of Psychology, Concordia University, Montreal, QC, Canada; i Virginia Commonwealth University Massey Comprehensive Cancer Center, Richmond, VA, USA; j University of Minnesota Medical School, Minneapolis, MI, USA; k The University of Texas MD Anderson Cancer Center, Houston, TX, USA

**Keywords:** Childhood cancer survivors, Symptom burden, Risky health behaviors, Healthcare utilization

## Abstract

**Background:**

Childhood cancer survivors face physical, psychological, and neurological symptoms that contribute to risky health behaviors and increased healthcare utilization. Traditional survivorship care models overlook risk associated with this symptom burden. The current study examined symptoms phenotypes to identify high-risk groups.

**Methods:**

Five-year survivors (N = 17,231; Mean [standard deviation] age = 27.4 [5.98]; 80% non-Hispanic White; 48% female) from the Childhood Cancer Survivor Study (NCT01120353) self-reported symptoms and risky behavior at baseline and first follow-up (original cohort data collection: baseline 1994–1998 and follow-up 2002–2004; expansion cohort: baseline 2008–2010 and follow-up 2014–2016). Medical records were extracted through chart review. Chronic health conditions (CHCs) were graded according to common terminology criteria for adverse events criteria. Latent class analysis derived symptom phenotypes.

**Findings:**

Five phenotypes emerged: 1) Low Burden (63.1%); 2) Cardio-Pulmonary-Pain (5.3%) 3); Neurologic-Pain (10.6%); 4) Psychological Distress-Pain (13.3%); 5) Global burden (7.7%). Compared to survivors with Low Burden, those in other symptom phenotypes were older, female, had lower education, no health insurance, smoked cigarettes, were physically inactive, and had ≥ grade 3 CHC (all ps < 0.05). Survivors in symptom phenotypes were at-risk for future emergency room use (all ps < 0.05). Risk for future physical inactivity was higher in Cardio-Pulmonary-Pain (OR = 1.19, CI = 1.09, 1.31), Global (OR = 1.12, CI = 1.02, 1.22), and Neurologic-Pain (OR = 1.18, CI = 1.10, 1.27) phenotypes. Cigarette use was higher in Cardio-Pulmonary-Pain (OR = 1.62, CI = 1.08, 2.42) and (Global OR = 1.65, CI = 1.17, 2.31) phenotypes.

**Interpretation:**

Symptom phenotyping identified groups at-risk for future risky health behaviors, which was not explained alone by diagnosis or CHCs. Integrating symptom assessments may guide interventions to improve health outcomes.

**Funding:**

The work was supported by the 10.13039/100000054National Cancer Institute (U24 CA055727, PI: GT Armstrong). Support to 10.13039/100007737St. Jude Children's Research Hospital was also provided by the National Cancer Institute Cancer Center Support grant (P30 CA021765, PI: CWM Roberts) and by the 10.13039/100012524American Lebanese Syrian Associated Charities.

**Research in context**

**Evidence before this study**

We searched PubMed, Google Scholar, and reviewed citations of relevant articles from database inception through early 2025 using the following terms: childhood cancer survivors, pediatric cancer survivors, symptom burden, chronic health conditions, psychological distress, patient-reported outcomes, survivorship care plans, healthcare use, healthcare utilization, emergency room use, and behavioral health interventions, psychological interventions. Existing survivorship care models primarily rely on treatment history and diagnosis to guide follow-up care but may not fully incorporate survivors’ current symptom burden which may influence future risky health behaviors and healthcare utilization. A phenotype-informed approach, one that clusters survivors based on shared patterns of perceived physical, psychological, and neurological symptoms may better identify high-risk individuals and inform tailored interventions.

**Added value of this study**

This study is the first to apply a phenotype-informed framework to a large, well-characterized cohort of adult childhood cancer survivors to examine long-term behavioral risk and healthcare utilization. Using latent class analysis, we identified five distinct symptom burden phenotypes, with nearly 40% of survivors classified into high-burden groups. These phenotypes were defined by co-occurring physical, psychological, and neurological symptoms and were predictive of future risky health behaviors, such as smoking and physical inactivity, as well as increased emergency healthcare use.

**Implications of all the available evidence**

By identifying survivors based on shared symptom experiences, this study supports the need for the development of tailored, multimodal interventions that address both symptom burden and behavioral risk, complementing standard follow-up care approaches. A phenotype-informed approach that integrates multidimensional symptom data alongside traditional risk-stratification methods may offer a more precise strategy for identifying survivors at elevated behavioral risk.

## Introduction

Childhood cancer survivors are at elevated risk for poor health behaviors (e.g., smoking, physical inactivity, poor diet) that are linked with persistent psychological, neurological, and physical symptoms.^1^, ^2^, ^3^ These behaviors not only contribute to the development and progression of chronic health conditions,^4^ but also increase the likelihood of emergency room visits and other unplanned healthcare utilization,^5^ exacerbating long-term health outcomes.^5^^,^^6^ While traditional survivorship care models emphasize treatment history and diagnosis to guide follow-up, these factors alone may not adequately predict which survivors are most vulnerable to maladaptive health behaviors or high healthcare utilization.^7^^,^^8^ These models do not account for survivor perspectives of current symptom burden, which may drive health behavior decisions,^9^ miss survivors at high risk for poor health outcomes who do not have high-risk treatment histories, and lack integration with behavioral and psychosocial risk factors.^8^ A phenotype-informed approach, that is, one that groups survivors based on shared patterns of perceived physical, psychological, and neurological symptoms may help identify survivors at high risk and inform tailored interventions that address symptom burden and future health behavior.

Identifying subgroups of survivors based on shared symptom experiences, a phenotype-driven approach, can support the development of tailored, multimodal interventions that align with each survivor's unique risk profile. This strategy reflects the shift toward personalized care models to optimize outcomes and resource allocation. In line with this framework, the current study leverages data from the Childhood Cancer Survivor Study to: (1) empirically identify latent classes (i.e., phenotypes) of symptom burden among long-term survivors; (2) examine cross-sectional associations between sociodemographic, diagnostic, and behavioral factors with symptom phenotypes; and (3) evaluate longitudinal associations between symptom phenotypes with future healthcare utilization and risky health behavior. Ultimately, this work aims to inform the design of precision interventions that can reduce long-term morbidity and improve quality of life for survivors.

## Methods

### Participants

The Childhood Cancer Survivor Study (CCSS) is a retrospectively identified cohort with longitudinal follow-up of ≥5 years survivors of childhood cancer, diagnosed before 21 years of age between 1970 and 1999, and recruited from 31 institutions across North America. Baseline assessments for the original cohort took place between 1994 and 1998 and follow-up assessments occurred between 2002 and 2004. The expansion cohort completed baseline assessments between 2008 and 2010 and follow-up assessments between 2014 and 2016. The cohort and methodology have been described elsewhere.^10^ In this analysis, participants were included if they were ≥18 years of age at baseline assessment (i.e., entry into CCSS) and completed a follow-up assessment.

### Measures

Symptom assessment was conducted at baseline and included 38 items from health and behavior questionnaires, including the Brief Symptom Inventory (BSI).^11^ Domains assessed included: sensation abnormalities (9 items); motor/movement difficulties (4 items); cardiac symptoms (3 items), pulmonary symptoms (2 items); pain symptoms (4 items); fatigue (2 items); gastrointestinal symptoms (1 item); memory difficulties (1 item); anxiety (6 items); and depression (6 items). Endorsement across any domain indicated the presence of symptoms, with the exception of depression and anxiety. Consistent with scoring practices for the BSI,^11^ each domain's score was converted to a T-score (mean of 50 and standard deviation of 10) with scores ≥63 indicating clinically elevated symptoms.

Demographics variables included education level, household income, insurance status, and marital status, collected via self-report at baseline. Sex assigned at birth, age, race/ethnicity, cancer diagnosis, and treatment exposures were abstracted from medical records at the treating institution.

Chronic health conditions (CHCs) were self-reported at baseline and follow-up. The severity of each condition was graded using the Common Terminology Criteria for Adverse Events (CTCAE version 4.3) system developed by the National Cancer Institute. Conditions were coded as absent (0), mild (grade 1), moderate (grade 2), severe/disabling (grade 3), or life-threatening (grade 4).^12^

Physical inactivity was assessed at baseline and follow-up using items from the Behavioral Risk Factor Surveillance Survey. Metabolic equivalent (MET) in hours per week were calculated and weighted by the standardized classification of energy expenditure (i.e., moderate and vigorous activity).^13^ Levels <9 MET-hours per week was the cutoff for physical inactivity as this aligns with established guidelines for adults.^14^ Body mass index (BMI) was calculated for participants at baseline and follow-up based on weight/height (kg/m^2^). BMI was categorized as with obesity (BMI≥30) or without obesity. Smoking cigarettes was assessed at baseline and follow-up via self-report on two questions related to current smoking (e.g., “Do you smoke now?”) and frequency of smoking (e.g., “Have you smoked at least 100 cigarettes in your entire life?”). Consistent with prior research,^15^ participants were categorized as “never smoker,” “former smoker,” and “current smoker.”

Participants reported healthcare utilization over the past two years. Responses were categorized into no healthcare, general health care (doctor's office), survivor-focused care (oncology cancer center), and long-term survivorship care (long-term follow-up clinic). Emergency room care was also coded dichotomously (yes vs. no emergency care).

### Statistical analyses

Latent class analysis empirically derived classes of symptom burden. Model fit criteria included the Bayesian information criterion (BIC), with lower BIC values indicating better model fit^16^; Vuong-Lo-Mendell-Rubin's Test^17^ for comparing model improvement between neighboring classes (e.g., 2 class solution vs. 3 class solution, 3 class solution vs. 4 class solution); and the entropy of the model. Multinomial logistic regression models with generalized logit link were used to examine associations between demographic, diagnosis, treatment exposures, CHCs, and baseline health behaviors with the derived latent classes. These models compared all symptom classes to a limited symptom class (i.e., Low Burden Group). Cross-sectional multivariable models examined associations between baseline latent class assignment and health utilizations and health behaviors, adjusting for demographic factors and relevant baseline behaviors. Multinomial logistic regression was used for categorical outcomes (healthcare type and smoking status); Poisson regression with robust variance estimation using generalized estimating equations (GEE) was used for binary outcomes (emergency room visits and physical inactivity). Consistent with prior symptom burden research, risk ratios >1.5 were considered clinically meaningful in increasing crease symptom burden risk,^18^ while the threshold for increasing risky health behaviors is lower (>1.16).^19^ Clinically meaningful results are presented in bold text below.

### Ethics statement

CCSS primary site IRB was approved by each of the 31 institutions (IRB Protocol # CR00007578, University of Minnesota), and informed consent was obtained prior to participants engagement in the study. The CCSS cohort study is registered with ClinicalTrials.gov (NCT01120353).

### Role of funding source

This work was supported by the National Cancer Institute (NCI; U24 CA055727, PI: GT Armstrong). Support to St. Jude Children's Research Hospital was also provided by the NNCI Cancer Center Support grant (P30 CA021765, PI: CWM Roberts) and by the American, Lebanese, Syrian Associated Charities (ALSAC). The NCI nor ALSAC were involved in the development of the study design, collection, analyses, or interpretation of the data. NCI and ALSAC were not involved in the drafting of the manuscript or the decision to submit the paper for publication.

## Results

Survivors (number [N] = 17,237) were 27.4 (standard deviation [SD] = 5.98) years of age at the baseline, identified as predominately male (52.3%), non-Hispanic White (80%), and represented a variety of cancer diagnoses (leukemia, 29.4%; CNS tumor 16.7%; Hodgkin lymphoma 16.1%; non-Hodgkin lymphoma 10.0%) (Table 1). Follow-up assessments were completed by 72.1% (N = 12,415) of survivors who completed baseline. Approximately 20% were lost to follow-up due to death. All cross-sectional analyses had minimal missingness (<5%), for which we employed complete case analysis. For the longitudinal analyses 40% of data was missing systematically by race/ethnicity. Non-Hispanic Black and Hispanic survivors were disproportionally more likely to not complete the follow-up assessment (Black Non-Hispanic: 58.8%, Hispanic: 55.3% vs. White Non-Hispanic: 36.7% dropout rates). To address potential bias from non-random missingness, we applied inverse probability weighting using race/ethnicity as the predictor on future health behaviors and healthcare utilization in longitudinal analyses.

**Table 1:** Demographics and medical information.

	N = 17,231
Sex	
% Female	47.6
% Male	52.3
Age at survey assessment	
Mean (Standard Deviation)	27.4 (5.98)
Age at diagnosis	
Mean (Standard Deviation)	
Race/Ethnicity	
% Hispanic	8.3
% Non-Hispanic Black	6.3
% Non-Hispanic White	80.0
% American Indian/Alaska Native (Non Hispanic)	0.5
% Asian or Pacific Islander (Non Hispanic)	1.5
% Unknown race and/or ethnicity	4.1
Diagnosis	
% Leukemia	29.4
% CNS Tumor	16.7
% Hodgkin's Lymphoma	16.1
% Bone Tumor	10.2
% Non-Hodgkin's lymphoma	10.0
% Wilms Tumor	7.6
% Neuroblastoma	5.0
% Soft Tissue Sarcoma	5.0
Treatment modalities	
Received radiation	
% Cranial	23.3
% Chest	22.0
% Abdomen	19.2
% Pelvic	15.4
Received chemotherapy	
% Alkylating agents	49.7
% Anthracycline	45.4
% Platinum agents	9.2
% Vinca alkaloids	60.8
% Retinoic acid	0.2
% Methotrexate	38.4
% Corticosteroids	39.8
Insurance status (% yes)	83.19
Income	
% ≤19,999	17.6
% $20,000–$39,999	21.7
% $40,000–$59,999	17.1
% Over $60,000	28.8
Marital status	
% Married	30.9
% Living as Married	7.8
% Widowed	0.0
% Divorced	3.2
% Separated or no longer living as married	4.5
Education level	
% 1–8 years	1.2
% 9–12 years	6.9
% Completed high school	20.0
% Training after high school	5.1
% Some college	31.5
% College graduate	26.4
% Post-graduate level	8.8

### Latent class analysis

**Fig. 1:**
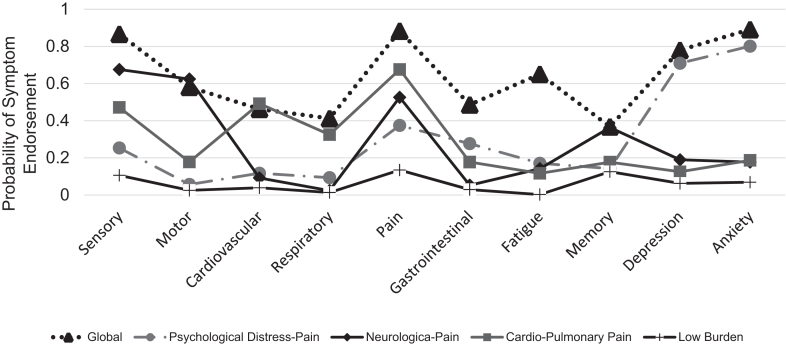
Latent classes of symptom burden across long-term survivors of the childhood cancer survivor study.

Latent class analysis supported a 5-class solution as the most parsimonious model (Fig. 1; Supplement Table S1). The largest class (63%) was the “Low Burden” phenotype, which evidenced a low probability of reporting any symptom. The “Cardio-Pulmonary-Pain” (5.3%) phenotype evidenced a high probability of reporting cardiac, respiratory, and pain symptoms. The “Neurologic-Pain” phenotype (10.6%) had a high probability of reporting sensory, motor, pain, and memory symptoms. The “Psychological Distress-Pain” phenotype (13.3%) evidenced a high probability of reporting pain and clinically significant depression and anxiety. The “Global” phenotype (7.7%) evidenced a high probability of reporting all symptoms.

### Baseline associations with symptom phenotypes

*Demographics* (Supplement Table S2). Increased risk of falling into a symptom phenotype, compared to the Low Burden phenotype, was associated with female sex (Psychological Distress-Pain Odds-ratio [OR] **1.52**, 95% Confidence Interval [CI] = 1.40, 1.65; Cardio-Pulmonary-Pain OR = **2.24**, CI = 1.96, 2.55; Neurologic-Pain OR = 1.42, CI = 1.29–1.56; Global OR = **1.86**, CI = 1.67, 2.07) older age at baseline assessment (Cardio-Pulmonary-Pain OR = 1.16, CI = 1.10, 1.22; Neurologic-Pain OR = 1.11, CI = 1.07, 1.16; Global OR = 1.18, CI = 1.13, 1.23), and older age at diagnosis (Psychological Distress-Pain OR = 1.01, CI = 1.00–1.02; Cardio-Pulmonary-Pain OR = 1.13, CI = 1.04, 1.22; Neurologic-Pain OR = 1.17, CI = 1.11, 1.25; Global OR = 1.28, CI = 1.19, 1.36).

*Sociodemographic Factors* (Supplement Table S3). Compared to Low Burden, elevated risk for symptom burden was associated with lack of health insurance (Psychological Distress-Pain OR = 1.23, CI = 1.10, 1.37; Global OR = **1.53**, CI = 1.34, 1.74), lower income (Psychological Distress-Pain OR = 1.33, CI = 1.21, 1.46; Cardio-Pulmonary-Pain OR = **1.52**, CI = 1.31, 1.76; Neurologic-Pain OR = **1.50,** CI = 1.34–1.67; Global OR = **1.99**, CI = 1.75, 2.26), and being unmarried/living with a partner (Psychological Distress-Pain OR = 1.21, CI = 1.10, 1.33; Neurologic-Pain OR = **1.64**, CI = 1.47–1.84; Global OR = 1.34, CI = 1.18, 1.52).

*Diagnoses* (Table 2). Compared to survivors diagnosed with Wilms tumor, survivors of bone tumor (OR = 1.33, CI = 1.07, 1.65), Hodgkin lymphoma (OR = 1.34, CI = 1.09, 1.64), and leukemia (OR = 1.21, CI = 1.01, 1.43) were at an increased risk of falling into Psychological Distress-Pain. Hodgkin lymphoma survivors had an increased risk of belonging to Cardio-Pulmonary (OR = 1.37, CI = 1.03, 1.82). Those with CNS tumors were at greatest risk for being assigned to Neurologic-pain (OR = **12.87**, CI = 9.49, 17.44).

**Table 2:** Multivariable analyses of primary childhood cancer diagnosis as predictor of symptom burden class.

Symptom class	Bone tumor	Central nervous system tumor	Hodgkin lymphoma	Leukemia	Neuroblastoma	Non-hodgkin lymphoma	Soft tissue tumor
	OR (95% CI)	OR (95% CI)	OR (95% CI)	OR (95% CI)	OR (95% CI)	OR (95% CI)	OR (95% CI)
Psychological distress-pain vs. low burden (reference)	**1.33 (1.07, 1.65)**	0.98 (0.79, 1.21)	**1.34 (1.09, 1.64)**	**1.21 (1.01, 1.43)**	1.21 (0.94, 1.55)	1.18 (0.95, 1.46)	0.91 (0.69, 1.19)
Cardio-pulmonary-pain vs. low burden (reference)	1.18 (0.86, 1.6)	**0.79 (0.58, 1.09)**	**1.37 (1.03, 1.82)**	**0.72 (0.56, 0.93)**	1.09 (0.76, 1.58)	1.01 (0.73, 1.39)	1.07 (0.74, 1.55)
Neurologic-pain vs. low burden (reference)	**2.86 (2.04, 4.01)**	**12.87 (9.49, 17.44)**	1.05 (0.74, 1.49)	**1.82 (1.34, 2.46)**	**2.58 (1.76, 3.77)**	**1.50 (1.05, 2.16)**	**2.06 (1.39, 3.05)**
Global vs. low burden (reference)	**2.33 (1.68, 3.24)**	**3.7 (2.73, 5.02)**	**1.89 (1.38, 2.6)**	**1.84 (1.38, 2.46)**	1.5 (1, 2.26)	**1.76 (1.25, 2.47)**	**2.23 (1.54, 3.22)**

**Note.** Wilm's tumor is reference group used across each diagnosis group. Model accounts for effects due participant sex and age at baseline. Bold font indicates statistical significance at least at p < 0.05. OR = odds ratio; CI = confidence interval.

*Chronic Health Conditions* (Supplemental Table S4). Survivors belonging to the Psychological Distress-Pain phenotype were at increased risk of having any grade 3 or higher CHCs, with the exception of hearing loss (OR range 1.13–1.63, all p-values<0.05). Survivors reporting symptoms associated with the Cardio-Pulmonary-Pain phenotype were at increased risk of having any grade 3 or higher CHC (OR range 1.36–3.02, all p-values<0.05). Survivors in the neurologic-pain phenotype were at increased risk for hearing loss (OR = **3.5**, CI = 2.93, 4.19), endocrine (OR = 1.25, CI = 1.12, 1.41), cardiovascular (OR = **1.52**, CI = 1.32, 1.75), and neurological CHCs (OR = **8.17**, CI = 7.27, 9.17). Survivors in the Global phenotype were at increased risk of having any grade 3 or higher CHC.

*Health Behaviors* (Table 3). Those who reported smoking at baseline compared to those who never smoked had increased risk of belonging to any symptom phenotypes (Cardio-Pulmonary-Pain OR = **1.90**, CI = 1.61, 2.24; Global OR = **3.42**, CI = 3.01, 3.88; Neurologic-Pain OR = 1.18, CI = 1.03, 1.35; Psychological Distress-Pain OR = **2.24**, CI = 2.02, 2.49) compared to the Low-Burden phenotype. Former smoking was a risk factor for both Global (OR = **1.54**, CI = 1.32, 1.80) and Psychological Distress-Pain (OR = 1.44, CI = 1.28, 1.62) phenotypes. Physical inactivity increased risk of belonging to several phenotypes (Cardio-Pulmonary-Pain OR = 1.47, CI = 1.28, 1.69; Global OR = 1.46, CI = 1.30, 1.63; Neurologic-Pain OR = 1.28, CI = 1.16, 1.41; Psychological Distress-Pain OR = 1.21, CI = 1.11, 1.32) compared to the Low-Burden class. Overweight/obesity was a risk factor for belonging to the Global (OR = 1.03, CI = 1.02, 1.04) and Neurologic-Pain (OR = 1.01, CI = 1.00, 1.02) phenotypes.

**Table 3:** Cross-sectional multivariable analyses of risky health behaviors at baseline predicting symptom burden class.

Symptom class	Smoking status (current vs. never)	Smoking status (former vs. never)	Physical inactivity (inactive vs. active)	Overweight/obesity (yes vs. no)
	OR (95% CI)	OR (95% CI)	OR (95% CI)	OR (95% CI)
Psychological distress-pain vs. low burden (reference)	**2.24 (2.02, 2.49)**	**1.44 (1.28, 1.62)**	**1.21 (1.11, 1.32)**	1.00 (0.99, 1.01)
Cardio-pulmonary-pain vs. low burden (reference)	**1.90 (1.61, 2.24)**	1.20 (0.99, 1.44)	**1.47 (1.28, 1.69)**	1.01 (1.00, 1.02)
Neurologic-pain vs. low burden (reference)	**1.18 (1.03, 1.35)**	0.86 (0.74, 1.00)	**1.28 (1.16, 1.41)**	**1.01 (1.00, 1.02)**
Global vs. low burden (reference)	**3.42 (3.01, 3.88)**	**1.54 (1.32, 1.80)**	**1.46 (1.30, 1.63)**	**1.03 (1.02, 1.04)**

**Note.** Models performed separately for each risky health behavior. Each model accounts for effects due participant sex and age at baseline. Bold font indicates statistical significance at least at p < 0.05. OR = odds ratio; CI = confidence interval.

### Symptom burden phenotypes and future health behaviors

*Healthcare Utilization* (Table 4). Adjusting for demographic, insurance status, and baseline CHCs, survivors with symptom burden phenotypes were more likely than the Low Burden phenotype to utilize emergency room care. Survivors in the Global phenotype were most likely to receive survivor-focused care as compared to general healthcare (oncology cancer center care OR = **1.29**, CI = 1.00, 1.675; long-term follow-up clinic OR = **1.29**, CI = 1.00, 1.67) and emergency healthcare (relative risk [RR] = **1.87**, CI = 1.67, 2.09) than those in the Low Burden phenotype. All burden phenotypes were at greater risk of emergency healthcare compared to the Low Burden phenotype (Cardio-Pulmonary-Pain RR = **1.49**, CI = 1.28, 1.72; Neurologic-Pain RR = **1.35**, CI = 1.20–1.51; Psychological Distress-Pain RR = **1.42**, CI = 1.28, 1.57).

**Table 4:** Longitudinal multivariable analyses of associations between symptom burden phenotype class at baseline predicting future health behaviors and healthcare utilization.

Risky health behavior	Psychological distress-pain vs. low burden	Cardio-pulmonary-pain vs. low burden	Neurologic-pain vs. low burden	Global vs. low burden
	Risk estimate (95% CI)	Risk estimate (95% CI)	Risk estimate (95% CI)	Risk estimate (95% CI)
Current smoker vs. non-smoker	1.18 (0.90, 1.54)	**1.62 (1.08, 2.42)**	1.185 (0.86, 1.63)	**1.65 (1.17, 2.31)**
Former smoker vs. non-smoker	**1.25 (1.00, 1.54)**	**1.57 (1.14, 2.16)**	1.06 (0.82, 1.36)	**1.46 (1.10, 1.95)**
Physical inactivity (inactive vs. active)	0.97 (0.9, 1.04)	**1.19 (1.09, 1.31)**	**1.18 (1.10, 1.27)**	**1.12 (1.02, 1.22)**
Healthcare utilization type				
No healthcare vs. general healthcare (reference)	0.82 (0.66, 1.02)	0.77 (0.53, 1.11)	1.22 (0.98, 1.53)	0.94 (0.70, 1.26)
Oncology cancer center care vs. general healthcare (reference)	0.92 (0.74, 1.13)	1.29 (0.97, 1.72)	0.92 (0.73, 1.16)	**1.29 (1.00, 1.65)**
Long-term follow-up vs. general healthcare (reference)	0.99 (0.81,1.21)	1.13 (0.84, 1.53)	**1.32 (1.07, 1.62)**	**1.29 (1.00, 1.66)**
Emergency room (Yes vs. No)	**1.42 (1.28, 1.57)**	**1.49 (1.28, 1.72)**	**1.35 (1.20, 1.51)**	**1.87 (1.67, 2.09)**

**Note.** Results from inverse probability weighted (IPW) multivariable models to address systematic dropout bias based on race/ethnicity. Models account for participant sex, age at baseline, race/ethnicity, health insurance status, baseline latent class assignment, and presence of baseline ≥ 3 chronic health conditions. Model performed separately for healthcare type, emergency room use, smoking status, and physical activity. Models for risky health behaviors (smoking, physical activity) additionally adjusted for baseline behaviors. Multinomial logistic regression was used for categorical outcomes (healthcare type and smoking status); Poisson regression with robust variance estimation using generalized estimating equations (GEE) was used for count/binary outcomes (emergency room visits and physical inactivity). Bold font indicates statistical significance at p < 0.05. Risk estimates are presented as odds ratio for healthcare type and smoking status, and relative risks for emergency room visits and physical inactivity. CI = confidence interval.

*Health Behaviors* (Table 4). Adjusting for demographic factors, insurance status, baseline chronic health conditions, and baseline health behavior, compared to the Low Burden profile survivors belonging to the Cardio-Pulmonary-Pain (OR = **1.62**, CI = 1.08–2.42) and (Global OR = **1.65**, CI = 1.17–2.31) demonstrated increased smoking behaviors at follow-up. Survivors belonging to the Global (OR = **1.46**, CI = 1.10–1.95), Cardio-Pulmonary-Pain (OR = **1.57**, CI = 1.14, 2.16), and Psychological Distress-Pain (OR = **1.25**, CI = 1.00–1.54) phenotypes were also more likely to report future former smoking behaviors. Several burden phenotypes (Cardio-Pulmonary-Pain RR = **1.19**, CI = 1.09–1.31; Global RR = 1.12, CI = 1.02–1.22; Neurologic-Pain RR = **1.18**, CI = 1.10–1.27) were also at greater future risk of physical inactivity compared to the Low-Burden class.

## Discussion

This study provides evidence that symptom burden phenotypes offer a clinically meaningful framework for understanding long-term behavioral risk and healthcare utilization among childhood cancer survivors. Nearly 40% of survivors were classified into high-burden phenotypes, each defined by co-occurring physical, psychological, and neurological symptoms. Notably, symptom phenotypes predicted future risky health behaviors, such as smoking and physical inactivity, as well as increased utilization of emergent healthcare services while adjusting for chronic health conditions and risky behavior at baseline. By identifying survivors based on shared symptom experiences, tailored, multimodal interventions that address both symptom burden and behavioral risk can be developed to meet survivors’ individual needs in conjunction with standard follow-up care approaches.

The Psychological Distress–Pain phenotype, the most prevalent burden profile in this study, exemplifies the need for integration of symptom assessment in conjunction with standard risk-stratification approaches as survivors in this group were not readily identified by the presence of CHCs or a single diagnostic category. Survivors in this phenotype experienced high levels of emotional distress and pain and were more likely to engage in risky health behaviors and utilize emergency services. These patterns may reflect limited access to effective coping strategies and mental health support. Prior research has documented cigarette use was more prevalent among those with chronic pain^20^ and psychological distress,^21^ and was used as a mechanism to support coping in non-survivorship populations. Further, individuals with depression and anxiety were nearly 2× as likely to visit the emergency room compared to individuals without depression and anxiety, with greater than a third reporting pain as the primary symptom.^22^ When individuals with psychological distress and pain were provided access to mental health services, emergency room utilization declined.^23^ While cognitive behavioral therapy and mindfulness-based interventions have demonstrated efficacy in supporting survivors,^24^^,^^25^ this type of care is often inaccessible for most cancer survivors.^26^ Thus, care navigation remains critical.

Other phenotypes, such as Cardio-Pulmonary–Pain and Neurologic–Pain, further illustrate the value of phenotype-based care planning. Survivors in the Cardio-Pulmonary–Pain group, most commonly diagnosed with Hodgkin lymphoma, demonstrated persistent engagement in risky health behaviors exacerbating their risk for cardiovascular disease.^27^ Similarly, the Neurologic–Pain phenotype, largely composed of CNS tumor survivors, showed high rates of physical inactivity, elevated BMI, and emergency room utilization, despite higher engagement in long-term follow-up care. These findings suggest that standard follow-up protocols may not adequately address the overlapping physical, neurological, and behavioral needs of these survivors, and that tailored interventions that include physical activity programs and behavioral health counseling may be particularly beneficial (See Supplemental Table S5).

The Global phenotype, characterized by elevated symptoms across all domains, represents a high-need group that is not easily identifiable based on clinical history alone. Survivors in this group exhibited high levels of healthcare utilization and risky behavior yet lacked clear diagnostic markers that would typically trigger enhanced follow-up. This underscores the importance of comprehensive, symptom-based assessments to identify survivors who may benefit from interdisciplinary, phenotype-informed care. Survivors in the Global phenotype may require integrated care models that combine mental health support, pain management, and behavioral health services.

Though there were unique differences across symptom phenotypes, there were several commonalities across all burden phenotypes that distinguished them from those with limited symptoms. One of the most striking findings from this study is that pain was a universal feature across all high-burden phenotypes, reinforcing its central role in the long-term survivorship experience. Prior research has identified upwards of 40% of survivors experience chronic pain.^25^ Despite its prevelance,^28^ pain remains underassessed and undertreated in survivorship care,^25^ and pharmacologic approaches including opioids have demonstrated limited efficacy among long-term childhood cancer survivors^28^ and raise significant health concerns for chronic pain populations.^25^ Given the significant number of survivors reporting pain symptomology as part of their phenotype, pain management strategies that include behavioral interventions as part of routine care are essential.^29^ It may also be beneficial for future research to explore how pain may present differently across phenotypes to inform phenotype-specific pain management support.

Risky health behaviors including smoking and physical inactivity were also consistently associated with all high-burden phenotypes, suggesting that these behaviors may be both a consequence of and contributor to persistent symptom burden. While prior studies have linked these behaviors to poorer quality of life and increased symptom severity,^30^^,^^31^ this study demonstrated that they are also predictive of phenotype membership, independent of cancer diagnosis or chronic health conditions. This underscores the importance of addressing behavioral risk not in isolation, but as part of a broader symptom management strategy. The bi-directional relationship between symptoms and behaviors, which are likely mediated by inflammatory and psychosocial mechanisms,^32^ suggests that interventions targeting both domains simultaneously may be more effective in reducing long-term morbidity.

Also notable across all burden profiles is that certain groups of survivors were disproportionately represented across high-burden phenotypes. Female sex and reduced access to resources (e.g., insurance, lower educational/income status) were consistently associated with increased odds of burden phenotype membership. Prior research has demonstrated female sex and resource status increase risk of pain symptomology in pediatric cancer survivors and may reflect challenges with accessing medical care,^33^ differences in pain related-coping,^34^ and disparities in pain treatment.^34^^,^^35^ These findings underscore the need for continued sensitivity to intersectional disparities that may impact childhood cancer survivors.

Despite the strengths of this research including the large cohort of survivors across multiple pediatric cancer treatment institutions assessed over several years, findings should be considered with several limitations. Apart from anxiety and depression, symptom severity was not captured as part of the symptom burden phenotype and these data were self-reported by presence of symptoms and not severity. In addition to presence of symptoms, symptom severity is likely to shape symptom phenotype clusters and differentially impact subsequent health behaviors, highlighting an important gap to address in future research. Similarly, while diagnostic factors were considered, treatment factors such as intensity of treatment were not examined, which likely contributes to disease burden. Further, though this population is diverse across many factors (e.g., diagnosis, geographical location, education), most participants were non-Hispanic White and non-White survivors were more likely to systematically dropout of follow-up visits. Prior research has documented survivors identifying as non-Hispanic Black and Hispanic were more likely to experience disparities in health outcomes including survivor-reported symptom burdens.^36^ Thus, symptom burden research may require a more targeted approach to better understand and support symptom management among this particularly vulnerable population. Finally, this research focused on long-term, adult survivors of childhood cancer. As treatment protocols improve and efforts to reduce symptom burden shift from intervention to prevention, it will be critical to consider younger populations earlier in the survivorship care continuum.

Childhood cancer survivors are at elevated risk for engaging in maladaptive health behaviors and increased healthcare utilization, both of which can compound long-term health challenges and tax community healthcare services. This study demonstrates that symptom burden phenotypes, defined by co-occurring physical, psychological, and neurological symptoms, predict future healthcare utilization and risky behavior, beyond other predictors like cancer diagnosis or chronic health conditions. Risky behaviors such as smoking and physical inactivity were consistently associated with high-burden phenotypes and may reflect attempts to cope with persistent symptoms like pain and emotional distress. These behaviors not only contribute to the progression of chronic conditions but also increase reliance on acute healthcare services.

## Contributors

RW conceptualized the study, drafted the manuscript, and led the editing process. DKS, LX, HD, and WL conducted the data analyses, verified the underlying data and results, and had full access to the study data throughout the analysis phase. MEM, TMB, NMA, KKN, BF, AKB, I–CH, GTA, RMH, DMG, and YY provided scientific input and oversight throughout the project. KRK contributed to study conceptualization, oversaw data analysis, and provided scientific editing and final manuscript review. GTA was responsible for funding acquisition and overall management of the CCSS study, including the provision of necessary resources. All authors read and approved the final version of the manuscript.

## Data sharing statement

The Childhood Cancer Survivor Study is a US National Cancer Institute funded resource (U24 CA55727) to promote and facilitate research among long-term survivors of cancer diagnosed during childhood and adolescence. CCSS data are publicly available on dbGaP at https://www.ncbi.nlm.nih.gov/gap/through its accession number phs001327.v2.p1, and on the St Jude Survivorship Portal within the St. Jude Cloud at https://survivorship.stjude.cloud/. Utilization of the CCSS data that leverages the expertise of CCSS Statistical and Survivorship research and resources will be considered on a case-by case basis. For this utilization, a research Application of Intent followed by an Analysis Concept Proposal must be submitted for evaluation by the CCSS Publications Committee. Users interested in utilizing this resource are encouraged to visit http://ccss.stjude.org. Full analytical data sets associated with CCSS publications since January of 2023 are also available on the St. Jude Survivorship Portal at https://viz.stjude.cloud/community/cancer-survivorship-community∼4/publications.

## Declaration of interests

DKS declares serving as a specialist grant reviewer for the Department of Defense (General Dynamics Information Technology); I–CH declares serving on the Research Advisory Board for Shriners Hospitals for Children; KRK declares serving on the Scientific Advisory Board for MD Anderson Cancer Center (Neuroscience Program); RMH declares NIH funding (R01CA261750; 3U24CA055727-29S1). All authors declare that they have no known competing financial interests or personal relationships that could have appeared to influence the work reported in this article.
