# Supplementary material for: Symptom burden, healthcare utilization, and risky behaviors in survivors of the childhood cancer survivor study (CCSS): an observation cohort study
Source: eClinicalMedicine. 2025 Nov 20;90:103657. doi: 10.1016/j.eclinm.2025.103657 (PMC12670949; doi:10.1016/j.eclinm.2025.103657)
Supplement: Supplementary Tables [file mmc1.docx]

Supplemental Table 1

| Model | BIC | Adjusted  BIC | VLMR*p*  *Compare with n-1 class* | Adjusted VLMR*p Compare with n-1 class* | Entropy |
| --- | --- | --- | --- | --- | --- |
| 2-class | 140678 | 140611 | <0.0001 | <0.0001 | 0.754 |
| 3-class | 139199 | 139097 | <0.0001 | <0.0001 | 0.723 |
| 4-class | 137842 | 137706 | <0.0001 | <0.0001 | 0.69 |
| 5-class | **137570** | **137399** | **<0.0001** | **<0.0001** | **0.708** |
| 6-class | 137547 | 137340 | 0.0004 | 0.0004 | 0.708 |
| 7-class | 137493 | 137252 | 0.1008 | 0.1023 | 0.691 |

| **Symptom Class** | **Sex**  (Female Vs. Male) | **Age at Baseline** | **Age at Diagnosis** | **Race/Ethnicity**  (Black, Non- Hispanic compared to White Non-Hispanic) | **Race/Ethnicity**  (Black, Non- Hispanic compared to White Non-Hispanic) |
| --- | --- | --- | --- | --- | --- |
|  | OR (95% CI) | OR (95% CI) | OR (95% CI) | OR (95% CI) | OR (95% CI) |
| **Low Burden (Reference)** | - | - | - | - | - |
| **Psychological Distress-Pain** | **1.52 (1.40, 1.65)** | 1.00 (0.97, 1.04) | **1.01 (1.00, 1.02)** | **0.71 (0.59, 0.85)** | 0.87 (0.75, 1.01) |
| **Cardio-Pulmonary-Pain** | **2.24 (1.96, 2.55)** | **1.16 (1.10, 1.22)** | **1.13 (1.04, 1.22)** | 1.11 (0.86, 1.42) | 1.08 (0.87,1.34) |
| **Neurologic-Pain** | **1.42 (1.29, 1.56)** | **1.11 (1.07, 1.16)** | **1.17 (1.11, 1.25)** | 0.86 (0.70, 1.05) | 0.86 (0.73,1.02) |
| **Global** | **1.86 (1.67, 2.07)** | **1.18 (1.13, 1.23)** | **1.28 (1.19, 1.36)** | 1.05 (0.85, 1.30) | 1.08 (0.90, 1.29) |

Supplemental Table 2.

Supplemental Table 3.

| **Symptom Class** | **Education**  <College vs. College Graduate | **Income**  <$40K vs. >$40K | **Marital Status**  Married/living with partner vs. other | **Health Insurance** |
| --- | --- | --- | --- | --- |
|  | OR (95% CI) | OR (95% CI) | OR (95% CI) | OR (95% CI) |
| **Low Burden (Reference)** | - | - | - | - |
| **Psychological Distress-Pain** | **1.18 (1.07, 1.3)** | **1.33 (1.21, 1.46)** | **1.21 (1.1, 1.33)** | **1.23 (1.1, 1.37)** |
| **Cardio-Pulmonary-Pain** | **1.85 (1.58, 2.16)** | **1.52 (1.31, 1.76)** | 0.90 (0.78, 1.04) | 1.00 (0.84, 1.2) |
| **Neurologic-Pain** | **1.55 (1.38, 1.73)** | **1.50 (1.34, 1.67)** | **1.64 (1.47, 1.84)** | **0.80 (0.7, 0.91)** |
| **Global** | **2.45 (2.12, 2.82)** | **1.99 (1.75, 2.26)** | **1.34 (1.18, 1.52)** | **1.53 (1.34, 1.74)** |

Supplemental Table 4.

| **Symptom Class** | **Any ≥Grade 3 Chronic Health Condition**  **(yes vs. no)** | **Secondary Malignant Neoplasm**  **(yes vs. no)** | **Hearing Loss**  **(yes vs. no)** | **Endocrine (yes vs. no)** | **Pulmonary**  **(yes vs. no)** | **Cardiovascular**  **(yes vs. no)** | **Neurological**  **(yes vs. no)** | **Gastrointestinal**  **(yes vs. no)** |
| --- | --- | --- | --- | --- | --- | --- | --- | --- |
|  | OR  (95% CI) | OR  (95% CI) | OR  (95% CI) | OR  (95% CI) | OR  (95% CI) | OR  (95% CI) | OR  (95% CI) | OR  (95% CI) |
| **Low Burden (Reference)** | - | - | - | - | - | - | - | - |
| **Psychological Distress-Pain** | **1.63**  **(1.34, 1.92)** | **1.34**  **(1.08, 1.65)** | 1.16  (0.93,1.45) | **1.13**  **(1.02, 1.25)** | **1.39**  **(1.15,1.67)** | **1.16**  **(1.02,1.33)** | **1.81**  **(1.58,2.06)** | **1.63**  **(1.39,1.92)** |
| **Cardio-Pulmonary-Pain** | **1.36**  **(1.06, 1.75)** | **1.34**  **(1.00, 1.80)** | **1.54**  **(1.14, 2.09)** | 1.11  (0.95,1.29) | **3.02**  **(2.46, 3.72)** | **2.76**  **(2.35, 3.24)** | **1.76**  **(1.45,2.14)** | **1.36**  **(1.06,1.75)** |
| **Neurologic-Pain** | 0.96  (.77, 1.19) | 0.99  (0.77, 1.28) | **3.50**  **(2.93,4.19)** | **1.25**  **(1.12, 1.41)** | **0.77**  **(0.60,0.99)** | **1.52**  **(1.32,1.75)** | **8.17**  **(7.27,9.17)** | 0.96  (0.77,1.19) |
| **Global** | **1.68**  **(1.37, 2.05)** | 0.96  (0.73,1.27) | **2.28**  **(1.84,2.83)** | **1.30**  **(1.14,1.47)** | **2.37**  **(1.95, 2.87)** | **1.97**  **(1.7, 2.27)** | **7.54**  **(6.63,8.57)** | **1.68**  **(1.37,2.05)** |

Supplemental Table 5.

| **Phenotype** | **Key Characteristics** | **Associated Risks** | **Suggested Interventions** |
| --- | --- | --- | --- |
| **Low Burden** | Few or no symptoms | Lower risk for poor health behaviors and healthcare use | Standard follow-up care |
| **Cardio-Pulmonary–Pain** | Sensory, cardiac, respiratory, and pain symptoms | Risk for smoking, physical inactivity, emergency room use | Standard follow-up care + exercise programs, heart health strategies, pain management |
| **Neurologic–Pain** | Sensory, motor, pain, and memory symptoms | Risk for inactivity, high BMI, emergency room use | Standard follow-up care + exercise programs, behavioral health counseling, pain management |
| **Psychological Distress–Pain** | Pain, depression, and anxiety | Risk for smoking, inactivity, emergency room use | Standard follow-up care + mental health support, integrated pain management, behavioral health counseling |
| **Global Burden** | Sensory, motor, pain, cardiac, respiratory, pain, depression, and anxiety symptoms | High healthcare use and behavioral risk | Comprehensive, interdisciplinary care targeting symptoms and behaviors |
